# Sensory evaluation of the bitterness of asenapine using D-sorbitol pretreatment: single-blind, placebo-controlled, crossover trial

**DOI:** 10.1186/s12888-023-04664-5

**Published:** 2023-03-14

**Authors:** Shuhei Wada, Kunihiro Iwamoto, Hiroki Okumura, Hirotake Hida, Shuichi Hiraoka, Aya Kamei, Daisuke Mori, Kiyofumi Yamada, Norio Ozaki

**Affiliations:** 1 Department of Psychiatry, Nagoya University Graduate School of Medicine, 65 Tsurumai, Showa, Nagoya, Aichi 466-8550 Japan; 2 Department of Neuropsychopharmacology and Hospital Pharmacy, Nagoya University Graduate School of Medicine, 65 Tsurumai-Cho, Showa-Ku, Nagoya, Aichi 466-8560 Japan; 3 Medical Affairs Department, Meiji Seika Pharma Co., Ltd, 2-4-16, Kyobashi, Chuo-Ku, Tokyo, 104-8002 Japan; 4 Brain and Mind Research Center, Nagoya University, 65 Tsurumai-Cho, Showa-Ku, Nagoya, Aichi 466-8550 Japan; 5 Pathophysiology of Mental Disorders, Nagoya University Graduate School of Medicine, 65 Tsurumai, Showa, Nagoya, Aichi 466-8550 Japan

**Keywords:** Asenapine, Adherence, D-Sorbitol pretreatment, Schizophrenia, Bitter taste of asenapine, Side effects, Maintenance therapy, Taste evaluation

## Abstract

**Background:**

Antipsychotics are essential in the acute treatment of and maintenance therapy for schizophrenia, but medication adherence and long-term treatment continuity are needed to maximize their effectiveness. Each antipsychotic has various side effects, which may affect adherence. Some patients with schizophrenia are reluctant to take asenapine because of its unique oral-related side effects, such as the bitter taste caused by sublingual administration. Our previous basic research found that D-sorbitol lowered the bitterness parameters of the taste sensors. However, whether D-sorbitol has the same effect in patients remains unclear. Therefore, using a D-sorbitol solution, we aim to evaluate changes in the bitterness of asenapine among patients with schizophrenia.

**Methods:**

In this single-blind, placebo-controlled, crossover trial, we plan to recruit 20 adult patients with schizophrenia spectrum disorder who take sublingual asenapine tablets. The participants will be divided into two groups (*n* = 10 each). Each group will be given a D-sorbitol or placebo solution on the first day for rinsing before taking the sublingual asenapine tablets. After a 1-day interval, the participants will rinse their mouths again with a different liquid. Questionnaires regarding changes in taste and the willingness to continue asenapine will be conducted before the start of the study and after each rinse. The primary and secondary end points will be a taste evaluation of bitterness, and the willingness to continue asenapine, respectively. Differences in questionnaire scores between the D-sorbitol and placebo solutions will be calculated and analyzed using a McNemar test.

**Discussion:**

This study aims to determine the efficacy of D-sorbitol in masking the bitter taste of asenapine. To our knowledge, it is the first intervention study using D-sorbitol for bitter taste of asenapine in patients with schizophrenia. Evidence of the efficacy of D-sorbitol could result in D-sorbitol pretreatment being an easy and inexpensive means of improving adherence to asenapine.

**Trial registration:**

This study was registered in the Japan Registry of Clinical Trials jRCTs041210019, on May 14, 2021. Ethics approval was obtained from the Nagoya University Clinical Research Review Board.

## Background

Antipsychotics are essential in the acute treatment of and maintenance therapy for schizophrenia. Maintenance of antipsychotic drugs not only prevents relapses and rehospitalizations, but also benefits patients in terms of quality of life [1]. All antipsychotics reduce overall symptoms more than do placebos, but they do not show clinically significant differences among drugs in terms of efficacy, except for clozapine [2]. Therefore, medication adherence and long-term treatment continuity are needed to maximize their effectiveness. In addition, the side effects of antipsychotics, especially metabolic side effects and those related to extrapyramidal symptoms, affect medication adherence [3].

Each antipsychotic has a unique safety profile that may affect adherence. Asenapine is the only sublingual antipsychotic approved (in 2009) by the United States Food and Drug Administration (FDA). A network meta-analysis of 32 oral antipsychotics reported that asenapine is effective against the positive, negative, and depressive symptoms associated with schizophrenia [2]. Several lines of evidence show that asenapine has minimal effect on metabolic side effects such as weight gain, glucose intolerance, and prolactin elevation [2, 4]. In addition, asenapine has been shown to be associated with a lower incidence of extrapyramidal side effects compared with other newer second-generation antipsychotics [5]. Although asenapine is associated with a lower risk of adverse events affecting medication adherence, it has characteristic side effects that may affect its tolerability.

Asenapine has unique oral-related side effects, such as the bitter taste caused by sublingual administration, so some patients with schizophrenia are reluctant to take it. For example, approximately 10% of patients reported experiencing oral hypoesthesia [6], and 6.5% of patients stopped taking their medication because of the bitter taste [7]. Asenapine is in a sublingual form because of its high first-pass effect. For this reason, patients should avoid eating or drinking for several minutes after administration to prevent a decrease in bioavailability [8]. Therefore, patients may need to tolerate the bitter taste of asenapine for a few minutes, which may affect adherence and compliance with the dosing procedure.

Reducing the bitter taste of pharmaceutical preparations is widely accepted as a way of improving patient adherence and compliance to a therapeutic drug regimen [9]. Dealing with the bitter taste of asenapine is critical for improving adherence. Our previous basic research showed that D-sorbitol lowered the bitterness parameters of the taste sensors [10]. D-Sorbitol is widely used in oral care products and has been approved as a pharmaceutical product, which makes it easy to use in a practical way. However, whether D-sorbitol has the same effect in patients remains unclear. Therefore, we decided to conduct a crossover study utilizing D-sorbitol as the primary endpoint to evaluate changes in the taste of asenapine in patients with schizophrenia. As a secondary endpoint, we will also conduct a survey on the willingness to continue asenapine.

## Methods

### Study design

The purpose of this single-blind, placebo-controlled, crossover study is to evaluate the efficacy of D-sorbitol in masking the bitter taste of sublingual asenapine tablets. D-Sorbitol oral solution 75% (KOWA, Aichi, Japan) will be used as the test solution in this study. The dosing period will be 1 day each in study periods ① and ②. Taste evaluations will be performed at home for outpatients and during hospitalization for inpatients, with a 1-day test interval to eliminate the carryover effect of D-sorbitol. Patients will be given an oral rinse with D-sorbitol or a placebo solution before taking the sublingual asenapine tablets. Then, after a 1-day interval, the patients will rinse their mouths again with a different liquid than that used on day 1. A questionnaire survey regarding taste evaluations and the intention to continue asenapine with the intervention will be conducted on the participants before the start of the study and after each rinse.

### Participants

Patients attending or hospitalized at the Department of Psychiatry, Nagoya University Hospital, Nagoya, Japan, will be included in the study. The sample size was set as 20 by the following statistical calculation. Using the 7-point Likert scale, we defined 1 to 3 points as improved and 4 to 7 points as non-improved. The McNemar test was used to estimate the sample size for the improved/non-improved binary data. McNemar’s test was used to determine the number of cases required to obtain a significant difference (significance level: 5% two-sided) with more than 90% power in the crossover study. As a result, the number of cases to be analyzed was nine for each group (18 cases in total), assuming a dropout rate of 10%. To reach the target sample size, we will recruit through advertisements at Nagoya University Hospital.

The inclusion criteria are as follows: 1. Adult patients with schizophrenia or psychotic features based on the Diagnostic and Statistical Manual of Mental Disorders, 5th edition (DSM-5), regardless of gender; 2. Patients using sublingual asenapine tablets for more than 2 weeks; and 3. Patients who can provide written informed consent. The exclusion criteria are: 1. Patients with physical diseases or symptoms requiring medical treatment; 2. Pregnant and lactating women; 3. Patients with a history of convulsions; 4. Patients with a substance use disorder; 5. Patients with a taste disorder; 6. Patients with an olfactory disorder; 7. Patients who cannot understand the questionnaire protocol; and 8. Other patients whose inclusion in the study is deemed inappropriate by the attending physician. We will interview participants to determine if they meet these exclusion criteria. The reason for excluding olfactory disorder is that an associations between olfactory and bitter taste receptors has been suggested, and olfactory disorder may also affect the evaluation of taste [11]. The discontinuation criteria are: 1. If the research participant requests discontinuation; and 2. If the attending physician determines that there will be a clear disadvantage in the participant’s disease.

### Randomization and blinding

The primary investigator or coinvestigators will enroll and assign the participants to the investigations. Two groups will be randomly assigned using the envelope method. D-Sorbitol and placebo will be administered in clear containers, as they are visually indistinguishable. The participants can distinguish between the two liquids based on their level of sweetness. We just informed the patients that we would ask them to evaluate the effect of the two different solutions on bitterness but did not indicate which flavor was the placebo. Even if they can distinguish between the liquids, blindness is expected to be maintained. Only the participants will be blinded.

### Study drug

Participants will receive D-sorbitol as the active drug and distilled water as the placebo. Participants will rinse with 25 mL of the study drug immediately before taking the sublingual asenapine tablet. Both D-sorbitol and distilled water will be kept clear and colorless to maintain blinding. During the study period, patients will be instructed to rinse their mouths by themselves at home or in the hospital ward. Afterwards, the patients will answer a multiple-choice questionnaire, which will be collected later.

### Experimental schedule

**Fig. 1:**
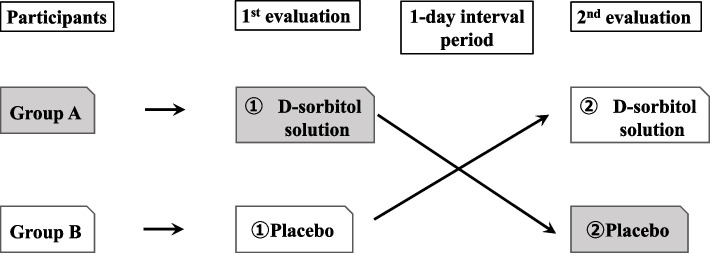
Experimental schedule. The study participants will rinse their mouths with 25 mL of D-sorbitol or placebo immediately before taking the sublingual asenapine tablet once a day. All participants will be randomly assigned to Group A or Group B (*n* = 10 each)

The experimental schedule is shown in Table 1 and Fig. 1. The study will have two groups. Group A will receive D-sorbitol first (test period ① using D-sorbitol and test period ② using placebo solution), and Group B will receive the placebo solution first (test period ① using placebo solution and test period ② using D-sorbitol). There will be a 1-day interval between study periods ① and ②. The study will be conducted in the ward for inpatients and at home for outpatients at the same time of day as the administration of the sublingual asenapine tablets. The participants will provide the following information: disease onset category, duration of illness from the first onset, DSM-5 specific terms, total Positive and Negative Syndrome Scale score at enrollment, sublingual asenapine tablet starting date, asenapine dosage, concomitant medications, comorbidities, smoking history, height, and weight. Before the study, the participants will complete a multiple-choice questionnaire regarding the side effects and duration of getting used to asenapine. Also, after the study is completed, a questionnaire regarding the efficacy of the intervention will be administered.

**Table 1:** Experimental schedule

	Experimental schedule				
	Baseline	Experimental period			Discontinuation
Day	Day 0	*Day 1*	*Day 2*	*Day 3*	
Participant background	○				
PANSS	○				
Height, weight	○				
Pre-questionnaire	○				
*Oral rinse with* *D-sorbitol or placebo*		○		○	
*Post-implementation questionnaire*		○		○	
*Adverse events*					○

*PANSS* Positive and Negative Syndrome Scale

### Questionnaire survey content

The pre-questionnaire will ask for a choice-type statement regarding the following items:

1. Length of time to get used to sublingual asenapine tablets
  Please circle the number that applies to how long it takes you to become accustomed to using asenapine sublingual tablets.
  ① No problem from the first time ② A few days ③ One week ④ Two weeks ⑤ One month ⑥ Not used to it yet
2. Time to become accustomed to the taste of sublingual asenapine tablets
  Please circle the number that applies to how long it took you to get used to the taste of asenapine sublingual tablets.
  ① No problem from the first time ② A few days ③ One week ④ Two weeks ⑤ One month ⑥ Not used to it yet
3. Presence or absence of adverse events before and after the use of sublingual asenapine tablets regarding the following items (weight gain, drowsiness, lightheadedness, somnolence, fatigue, insomnia, hand tremors, difficulty moving the body, dry mouth, increased appetite, constipation, dizziness, decreased libido, galactorrhea, menstrual irregularities, and akathisia)

Each question will be rated on a 6-point scale, except for question 3 on the pre-questionnaire.

The post-implementation questionnaires will ask for a choice-type statement regarding the following:

1. Oral condition
  Please describe the condition of your mouth before rinsing today.
  ①Very dry ② Feels dry ③ Normal ④ Concern about saliva ⑤ Very much saliva
2. Change in bitter taste when taking sublingual tablets
  How did you feel the bitterness of this sublingual tablet compared to the bitterness you used to feel?
  ①Almost no bitterness ② Bitterness has been clearly reduced ③ Bitterness seems to have decreased a little ④ No change ⑤ Bitterness has become a little stronger ⑥ Bitterness has become obviously stronger ⑦ Bitterness has become so strong that it is difficult to use
3. Whether the taste of asenapine with the intervention is easy to continue
  Do you feel that the taste is easy to continue using?
  ①Taste that you can continue to use without any problem ②Easier to continue using than before ③Same taste as before ④Taste more difficult to continue using than before ⑤Difficult to continue using
  All questionnaires will be conducted in Japanese.

### Primary outcome

The primary end point is the taste evaluation of bitterness, which addresses post-implementation question 2. The participants will self-assess on a 7-point scale regarding the use of D-sorbitol and the reduction of the bitter taste.

### Secondary outcome

The secondary end point will be the willingness to continue asenapine, subjectively self-rated on a 5-point scale, which addresses post-implementation question 3. The use of mouthwash before taking asenapine is expected to reduce its bitterness. There is expected to be a need to evaluate whether the reduction of bitterness affects adherence to asenapine.

### Statistical analysis

The primary endpoint was evaluated on a 7-point Likert scale, with 1 to 3 points defined as improvement and 4 to 7 points as non-improvement. The secondary endpoint was evaluated on a 5-point Likert scale, with 1 to 2 points defined as improvement and 3 to 5 points as non-improvement. The difference in population proportions for improved/non-improved binary data will be analyzed using a McNemar test. Participants with missing data will be excluded from the analysis, and no interim analyses will be performed.

### Adverse events

The D-sorbitol and placebo solutions are considered safe for humans. In the case of an adverse event, the participant may be withdrawn from the study at the discretion of the attending physician. At that time, the participant will receive appropriate medical attention. Adverse events will be monitored and recorded one by one. The adverse event incidence rates will be calculated, but not aggregated or analyzed.

### Ethics and dissemination

This study was registered in the Japan Registry of Clinical Trials (jRCTs041210019) on May 14, 2021. The study protocol was approved by the Nagoya University Clinical Research Review Board (CRB4180004). All methods will be carried out in accordance with the Declaration of Helsinki. Informed consent will be obtained from all study participants by the primary investigator or coinvestigators. For privacy protection, all participants will be identified using an anonymous identification code. The participants will be informed by the researcher about potential benefits, risks, alternatives, and responsibility during the study throughout the consent process. If any necessary experimental data are provided to a joint research institution, it will be carefully protected using only the participant’s identification code and a corresponding table. The acquisition of informed consent, the inclusion/exclusion criteria, participant eligibility, and the occurrence of any adverse events will be confirmed by an independent monitor from the research institution. The monitor will check whether the research procedure was carried out based on the approved procedure and confirm that the data storage method is appropriate. The findings from the research will be published in peer-reviewed journals and presented at local, national, and international conferences.

### Consent to publish

Not applicable.

## Discussion

This purpose of this study is to determine the efficacy of D-sorbitol in masking the bitter taste of asenapine. To our knowledge, this is the first intervention study with using D-sorbitol in patients with schizophrenia. There have been reports from asenapine clinical trials regarding the bitter taste of asenapine. The FDA has approved a black cherry-flavored sublingual tablet [12]. Although a registered randomized controlled trial compared raspberry-flavored with unflavored asenapine, its outcome has yet to be published [12, 13]. Even if this flavored formulation can reduce the bitterness of asenapine, it is still not available outside the United States. The clinical data show that the frequency of oral hypoesthesia with asenapine in Western populations may differ from that in Asian populations. For example, the incidence of oral hypoesthesia in Asian populations is about 10%, compared with only about 5% in Western populations [6, 13]. This gap may be the result of differences in the perceived intensity of the bitter taste among ethnic groups. Asians are far more sensitive to bitter tastes than are African-Americans, White people, and Hispanics [14].

Although D-sorbitol is more effective in masking bitterness in basic research, other substances, such as glycerin, propylene glycol, saccharin, and xylitol, can mask the bitter taste of asenapine [10]. However, D-sorbitol is inexpensive, available by prescription, and can be used without concerns about drug interactions or side effects, making it easy to put into practical use. It also offers significant advantages for patients who already comply with the administration procedure of sublingual asenapine tablets, such as the ability to alleviate side effects without changing drugs. If this trial can confirm the efficacy of D-sorbitol, pretreatment with D-sorbitol could provide an easy and inexpensive means of improving adherence.

In 2019, the FDA approved an asenapine formulation that may improve adherence. However, other factors may affect adherence. There is concern that compliance will decline because of economic reasons, i.e., its high price [15–17]. The use of an asenapine transdermal patch may be complicated for some patients, and may lead to erythema at the application site as a distinctive side effect; this was reported in approximately 10% of cases compared with a placebo in a phase III trial [18]. Furthermore, the asenapine transdermal patch differs significantly from sublingual asenapine tablets in terms of having an immediate effect on agitation [19, 20]. However, there are still situations in which the use of sublingual tablets is appropriate [21], and more treatment options could be beneficial for shared decision-making between psychiatrists and patients.

This study has several limitations. Our investigation does not include participants who discontinued asenapine owing to bitter taste, hence the evaluation of D-sorbitol could change if the sample population changes. Another limitation is that this study does not evaluate oral hypoesthesia as a parameter because bitterness is the main target of evaluation. Inclusion of patients newly taking asenapine and evaluation of oral hypoesthesia are needed in future studies. If this study can clarify the effect of D-Sorbitol on the bitterness of asenapine, it will be helpful to both patients and doctors as one way to improve adherence to asenapine.

## Data Availability

Not applicable.

## Abbreviations

DSM-5: Diagnostic and Statistical Manual of Mental Disorders, 5th edition
FDA: Food and Drug Administration
PANSS: Positive and Negative Syndrome Scale

## References

[1] Ceraso A, Lin JJ, Schneider-Thoma J, Siafis S, Heres S, Kissling W, Davis JM, Leucht S (2022). Maintenance Treatment With Antipsychotic Drugs in Schizophrenia: A Cochrane Systematic Review and Meta-analysis. Schizophr Bull.

[2] Huhn M, Nikolakopoulou A, Schneider-Thoma J, Krause M, Samara M, Peter N, Arndt T, Bäckers L, Rothe P, Cipriani A (2019). Comparative efficacy and tolerability of 32 oral antipsychotics for the acute treatment of adults with multi-episode schizophrenia: a systematic review and network meta-analysis. Lancet.

[3] Dibonaventura M, Gabriel S, Dupclay L, Gupta S, Kim E (2012). A patient perspective of the impact of medication side effects on adherence: results of a cross-sectional nationwide survey of patients with schizophrenia. BMC Psychiatry.

[4] Greger J, Aladeen T, Lewandowski E, Wojcik R, Westphal E, Rainka M, Capote H (2021). Comparison of the Metabolic Characteristics of Newer Second Generation Antipsychotics: Brexpiprazole, Lurasidone, Asenapine, Cariprazine, and Iloperidone With Olanzapine as a Comparator. J Clin Psychopharmacol.

[5] Chow CL, Kadouh NK, Bostwick JR, Vandenberg AM (2020). Akathisia and Newer Second-Generation Antipsychotic Drugs: A Review of Current Evidence. Pharmacotherapy J Human Pharmacol Drug Ther.

[6] Kinoshita T, Bai Y-M, Kim J-H, Miyake M, Oshima N (2016). Efficacy and safety of asenapine in Asian patients with an acute exacerbation of schizophrenia: a multicentre, randomized, double-blind, 6-week, placebo-controlled study. Psychopharmacology.

[7] Matsuzaki H, Hatano M, Iwata M, Yamada S (2021). Treatment Continuation of Asenapine or Olanzapine in Japanese Schizophrenia Patients: A Propensity Score Matched Study. Neuropsychiatr Dis Treat.

[8] Citrome L (2014). Asenapine review, part I: chemistry, receptor affinity profile, pharmacokinetics and metabolism. Expert Opin Drug Metab Toxicol.

[9] Beltrán LR, Sterneder S, Hasural A, Paetz S, Hans J, Ley JP, Somoza V (2022). Reducing the Bitter Taste of Pharmaceuticals Using Cell-Based Identification of Bitter-Masking Compounds. Pharmaceuticals.

[10] Kaneshige J, Kon M, Kai N, Hiraoka S, Ohta M, Ozaki N (2020). Evaluation of Taste-masking Effect of Asenapine Maleate Using Ingredients for Oral Care Products by Taste Sensor. J New Remed Clin.

[11] Alfonso-Prieto M (2021). Bitter Taste and Olfactory Receptors: Beyond Chemical Sensing in the Tongue and the Nose. J Membr Biol.

[12] Citrome L (2014). Asenapine review, part II: clinical efficacy, safety and tolerability. Expert Opin Drug Saf.

[13] Citrome L (2009). Asenapine for schizophrenia and bipolar disorder: a review of the efficacy and safety profile for this newly approved sublingually absorbed second-generation antipsychotic. Int J Clin Pract.

[14] Choi SE, Chan J (2015). Relationship of 6-n-propylthiouracil taste intensity and chili pepper use with body mass index, energy intake, and fat intake within an ethnically diverse population. J Acad Nutr Diet.

[15] Asenapine [https://www.goodrx.com/asenapine] Accessed 18 Dec 2022.

[16] Secuado [https://www.goodrx.com/secuado] Accessed 18 Dec 2022.

[17] Shuler K (2014). Approaches to improve adherence to pharmacotherapy in patients with schizophrenia. Patient Prefer Adherence.

[18] Citrome L, Walling DP, Zeni CM, Starling BR, Terahara T, Kuriki M (2020). Park; AS, Komaroff M: Efficacy and Safety of HP-3070, an Asenapine Transdermal System, in Patients With Schizophrenia. J Clin Psychiatry.

[19] Pratts M, Citrome L, Grant W, Leso L, Opler LA (2014). A single-dose, randomized, double-blind, placebo-controlled trial of sublingual asenapine for acute agitation. Acta Psychiatr Scand.

[20] Suzuki K, Castelli M, Komaroff M, Starling B, Terahara T, Citrome L (2021). Pharmacokinetic Profile of the Asenapine Transdermal System (HP-3070). J Clin Psychopharmacol.

[21] Musselman M, Faden J, Citrome L (2021). Asenapine: an atypical antipsychotic with atypical formulations. Ther Adv Psychopharmacol.

